# Population-Sequencing as a Biomarker of *Burkholderia mallei* and *Burkholderia pseudomallei* Evolution through Microbial Forensic Analysis

**DOI:** 10.1155/2013/801505

**Published:** 2013-12-17

**Authors:** John P. Jakupciak, Jeffrey M. Wells, Richard J. Karalus, David R. Pawlowski, Jeffrey S. Lin, Andrew B. Feldman

**Affiliations:** ^1^Cipher Systems, 2661 Riva Road, Annapolis, MD 21401, USA; ^2^CUBRC, Buffalo, NY 14225, USA; ^3^The Johns Hopkins University, Applied Physics Laboratory, 11100 Johns Hopkins Road, Laurel, MD 20723, USA

## Abstract

Large-scale genomics projects are identifying biomarkers to detect human disease. *B. pseudomallei* and *B. mallei* are two closely related select agents that cause melioidosis and glanders. Accurate characterization of metagenomic samples is dependent on accurate measurements of genetic variation between isolates with resolution down to strain level. Often single biomarker sensitivity is augmented by use of multiple or panels of biomarkers. In parallel with single biomarker validation, advances in DNA sequencing enable analysis of entire genomes in a single run: population-sequencing. Potentially, direct sequencing could be used to analyze an entire genome to serve as the biomarker for genome identification. However, genome variation and population diversity complicate use of direct sequencing, as well as differences caused by sample preparation protocols including sequencing artifacts and mistakes. As part of a Department of Homeland Security program in bacterial forensics, we examined how to implement whole genome sequencing (WGS) analysis as a judicially defensible forensic method for attributing microbial sample relatedness; and also to determine the strengths and limitations of whole genome sequence analysis in a forensics context. Herein, we demonstrate use of sequencing to provide genetic characterization of populations: direct sequencing of populations.

## 1. Introduction

Genome sequencing data of mixtures can function as biomarkers for identification of genetic content of samples and to establish a sample's genome profile, inclusive of major and minor genome components, drill down to identify SNPs and mutation events, compare relatedness of genetic content between samples, profile-to-profile, and provide a probabilistic or statistical scoring confidence for sample attribution. While high-throughput, automated sequencing has been used for years, analysis of sequencing information has focused on consensus sequencing [1–5]. In addition, sequencing has been used to infer microbial relationships [6–8]. Due to the ease of generating large volumes of sequence data, there has been pressure to develop computational tools [9]. Novel approaches, based on probabilistic analysis of sequencing information for mixtures and metagenomic samples, enable a broad capture of sequence data from a single run to characterize multiple genomes in a sample, even in isolates that are considered pure [10, 11]. When identifying genomes and determining the distribution of related organisms, knowing the populations of genomes in a sample is critical to accurate biomarker detection of disease, especially to the strain-level of identification.

In this study, we investigated the capability of whole genome sequence analysis to characterize relatedness/association between closely related populations of known ancestry under controlled conditions in the laboratory. Accompanying goals were to verify whether whole genome sequencing analysis is a reliable microbial forensic method for attributing relatedness, characterizing the extent of evolutionary mutations of *Burkholderia mallei* (glanders) and* Burkholderia pseudomallei* (melioidosis) populations over time, and understanding the strengths and limitations of whole genome sequence analysis in a forensics context. *Burkholderia pseudomallei* is the causative agent of melioidosis. The 7.2 Mb genome of *Burkholderia pseudomallei* consists of two chromosomes of 4,074,542 bp and 3,173,005 bp [12, 13].

Overall, the knowledge of this pathogen and its disease is limited. Pulsed field gel electrophoresis (PFGE) and MLST have been the current standard for identifying strains of *Burkholderia*; however, multilocus variable number tandem repeat (VNTR) and MLVA methods have been introduced [14–16]. The calculated mutation rate is 9.8 × 10^−5^ mutations/generation, which is slightly greater than *Y. pestis* and *E.coli*O157 [17].

*Burkholderia mallei* is rarely associated with human infection, and is more commonly seen in domesticated animals such as horses, donkeys, and mules where it causes glanders. The *B. mallei* genome has undergone a substantial genome reduction since the last common ancestor with *B. pseudomallei*, facilitated apparently by recombination events at the highly identical copies of transposase genes that are widespread in the *B. mallei* genome. The pathogen is hostadapted and is not found typically in the environment outside of its host [18]. Glanders is often fatal if not treated with antibiotics, and transmission can occur through the air, or more commonly when in contact with infected animals. Rapid-onset pneumonia, bacteremia (spread of the organism through the blood), pustules, and death are common outcomes during infection. The virulence mechanisms are not well understood [19, 20].

The Gram-negative pathogen *B. pseudomallei* is often fatal disease of both humans and animals [21]. Endemic to parts of South East Asia and Northern Australia, it is considered a major tropical pathogen and Category B biowarfare agent [22, 23]. In many countries, *B. pseudomallei* can only be experimentally manipulated under biosafety level 3 (BSL3) conditions.

### 1.1. Background of Microbial Forensics

Determining the source population of a biothreat agent attack is a goal of microbial forensics. Forensic scientists attempt to uncover DNA evidence to pinpoint a suspect and verify evidence for proof of guilt that will hold up in court. Microbial forensics played a major role in the Amerithrax investigation following the 2001 anthrax attacks on members of Congress and the public [24]. The utility of direct sequencing analysis for characterization of sample populations is a new paradigm for genome identification [11]. Recent investigations have shown strategies to process high-throughput DNA sequencing for accurate identification of major and minor populations and their concentrations [10].

For many years, microbial forensics relied heavily on amplified fragment length polymorphism (AFLP) and polymerase chain reaction (PCR) methods to attribute “relatedness” between crime scene evidence and suspected source samples [25]. After validation of hundreds of thousands of probes, high density microarrays can detect expected genomes, based on probe selection [26]. Since the emergence of next generation sequencing platforms within the last few years, genomic analysis of whole genome data is gaining popularity as a forensic technique. Whole genome sequencing provides vastly more quantity and specificity than either PCR or AFLP, and the implementation, reliability, and limitations of microbial genomic analysis need to be optimized, validated, and understood.

A number of studies combined AFLP, restriction fragment length polymorphism (RFLP), and PCR techniques for better discrimination between bacterial pathogen strains. AFLP involves cleaving sample genomic DNA with two or more restriction enzymes at base specific positions, then analyzing changes in DNA fragment lengths between different samples [27–29]. Changes in fragment length indicate changes in base identities or indels at the points where the restriction enzymes cleave the genomic DNA. Each fragment length change becomes a biomarker that may be compared with other strain biomarkers to construct a phylogenetic relationship among the separate populations.

Early PCR analysis of pathogenic bacteria was achieved by as few as 2–4 primers 20–24 nt in length [30]. As PCR techniques advanced, multiplexing primers were used to analyze various known static biomarkers that discriminate between different microbial pathogens [31]. Modern PCR arrays can achieve over 100 fold oligomer target specificities [32]. Even 100-fold multiplexing of oligomer targets represents a small proportion of the available number of biomarkers that can be detected with WGS. Furthermore, only short, predetermined primer sequences are detected with PCR, requiring prior knowledge about the sample for satisfactory primer selection. In addition some targets are shared across species. For example, screening of O-antigen genes showed the presence of multiple O-antigen types and sero-cross reactivity in near-neighbor species [33].

On the other hand, PCR amplifies minute amounts of DNA within a much larger background signal. Thus, rare variant polymorphisms or low concentration pathogens may be detected with PCR, if the right primers are available. Due to its amplification ability in the presence of high background, long PCR amplicons will likely continue to have a purpose in microbial forensic analysis for the detection and validation of rare variant genomes mixed in populations [34]. The limitations of conventional methods have been reviewed elsewhere. The focus of this paper is on the use of sequencing to characterize genome populations in a sample. Population-sequencing is an economic way of assessing species diversity that provides resolution at the strain level [35–40].

The first bacterial genome sequences were published in 1995, and within 15 years, over a thousand fully sequenced bacterial genomes have become publicly available [41].

Using sequencing to measure relative populations to identify simultaneously the organisms present in microbial populations with respect to the specific taxa (e.g., genus, species, subspecies, and strain) of bacteria, viruses, parasites, fungi, or nucleic acid fragments including plasmids and mobile genomic components requires bioinformatics tools to accurately match sequencing output. The greatest limitation of WGS is the inherent machine errors arising from the DNA sequencing process. Commonly reported sequencing error rates range from 0.1% for Illumina sequencers to 15% (single pass error) for PacBio single molecule sequencers [42]. Even at the level of a 0.1% error rate, the actual presence of a sequence variation in sample data must be verified statistically. For example, for a genome maintained in GenBank that has approximately 3 million base pairs, even, assuming, the best case scenario sequencing error rate of 0.1%, will generate 3,000+ errors spread throughout the entire genome [43].

Erroneous sequences appear identical to true variations within the data. Therefore, variant validation, or a calibrant [11] is required before trusting sequence variations reported by the sequencer. Stringency conditions applied to variant validation include

- variants must appear in both forward and reverse sequenced reads,
- minimum percentage of consensus,
- minimum number of occurrences in agreement,
- base calling quality score.

To distinguish between errors and true positive calls, Bayesian statistics are applied to calculate posterior probabilities for the validity of each variant detected. Optimally, each criterion can be used to enhance or diminish the probability for each variant's trustworthiness. In addition, given the rate of genomic mutation and growing evidence of horizontal gene transfer, static methods that rely on predetermined signatures produce false negative results if (a) mutation has occurred in the nucleic acid sequence of the sample relative to signatures, (b) the target signature was horizontally transferred, or (c) genomic near neighbors are present in the sample.

Our approach involved a combination of alignment and alignment-free comparisons of sequence information. Our genomic sequence data involved massive amounts of relatively short reads (~100 nt). For genome assembly, it would be more ideal to sequence sample DNA with a platform that provides long read or paired end data for better assembly of large (supercontigs). Several groups, including our own, also utilize unaligned, unassembled sequence approaches to analyze genomic data [44]. Advantages of unaligned approaches include

- the ability to examine all reads rather than the subset of aligned reads,
- faster results that require less expert analysis,
- more straightforward scoring for genome matches.

On the other hand, aligning reads against reference genomes enables the extraction of genomic details not available from unaligned approaches. Such details include

- longer length matches to reference sequences,
- varying gene recombinations and indels within the genome,
- better discrimination between similar sequences,
- determination of coverage depth and percent genome coverage,
- more accurate detection and validation of polymorphisms.

## 2. Materials and Methods

### 2.1. Experimental Design

The purpose of this experiment was threefold as follows.

1. Observe DNA sequence mutations arising from an originally clonal isolate of *Burkholderia mallei* strain China 7 (ATCC 23744), and *Burkholderia pseudomallei* strain 668 when cultured under stressful conditions in the laboratory.
2. Determine the strengths and limitations of whole genome sequence analysis for characterizing variation between similar substrains.
3. Advance methods for determining “relatedness” between microbial samples.

In the forensics use case, evolutionary pressure and manipulation in the laboratory can be measured to assess dynamics of bacterial metapopulations. We incorporated stress in our experiments because we wanted enough variation under laboratory conditions to compare lineages to source to assess WGS utility in attribution. For example, samples maintained under controlled conditions, laboratory stocks, may show less diversity than samples found in nature. Instead of analysis at a few stable sites, we evaluated analysis across the entire genome and to use the entire genome as a biomarker to build genome population content (context) profiles of samples to maximize data for microbial forensics to establish statements regarding the relationship of manipulated samples and reference samples.

The sequences were from a single colony, which corresponds to a single constituent within the parent population. Generally, during growth, this bacterium will accrue new random, and fitness-selected mutations, as well as rearrangements that result in consensus and population differences in the sequence content with respect to the reference genome.

**Figure 1:**
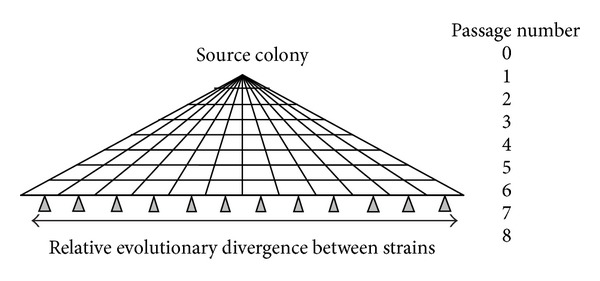
A schematic diagram of the experimental design. Theoretical accumulation of mutational variations among the 12 bacterial culture lineages.

A single colony of *Burkholderia pseudomallei and Burkholderia mallei* was passaged into 12 different plates. These twelve bacterial cultures were maintained separately over the course of seven more passages. Each culture passage was started with a single clonal colony. This created a single genome bottleneck at each passage step. Mutational variations differentiating each lineage were thus a result of initial variation in the source clonally derived culture plus mutations accumulated during the course of the eight growth and passage steps (Figure 1).

**Figure 2:**
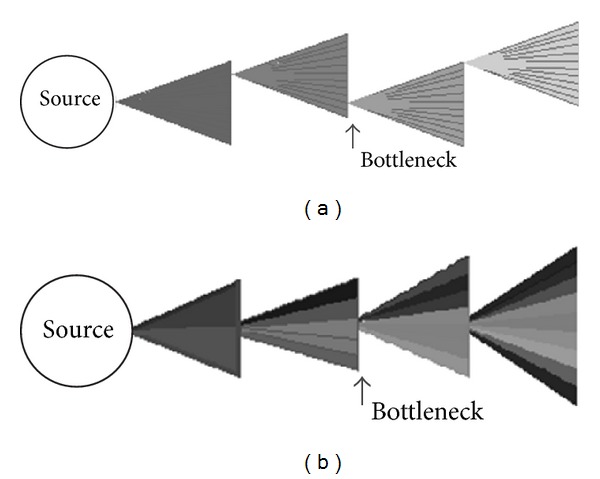
Accumulated genomic diversity expected from different passaging approaches. (a) Imposing a single cell genetic bottleneck at each passage step causes a gradual mutational shift with all descendent cells being closely related to one another. (b) By passaging a random subset of microbes at each step, accumulated mutational diversity within the lineage population is expected to be much greater.

There are two approaches that may be taken relative to the genetic bottlenecks at each passage step. The first approach, which we chose for this experiment, involved reducing all intralineage variation down to approximately zero by selecting only one clonal colony for passaging. The second approach would be to randomly select a subset of cells from the flask for subsequent passage. This approach should theoretically maintain a higher level of genetic diversity among the “lineage,” since not all microbes would be related to each other within each lineage at the end of the experiment. As genetic mutations accumulate over many generations, each lineage theoretically should become more diverse within itself; however, many of the cells within the same lineage would be no more related to each other than they would be to microbes in another lineage (Figure 2).

At the end of eight passages, six clones from each of the twelve lineages were collected. DNA was isolated from each clone and sequenced using an Illumina Genome Analyzer IIx platform as follows:

- single ended,
- average read length,
- other read statistics.

### 2.2. Culturing Media and Methods

**Figure 3:**
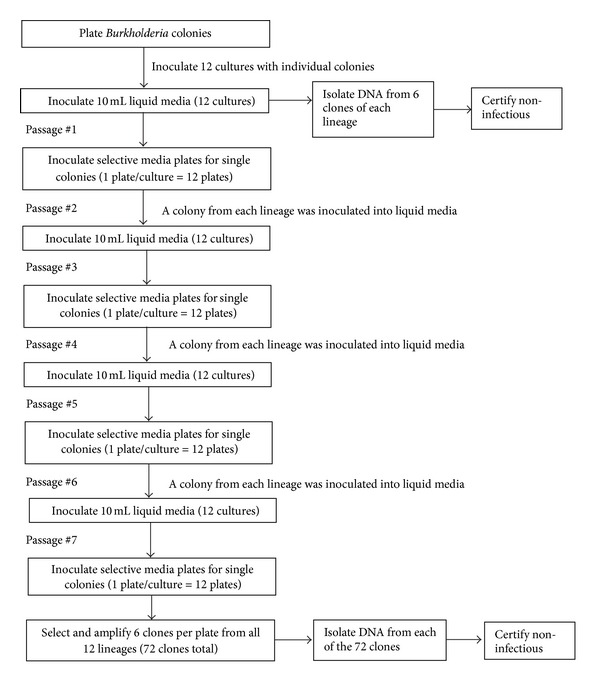
Pathogen culturing protocol in selective media. Following the seventh passage step, six (6) colonies from each of the twelve cultures were selected, amplified in liquid media, and the DNA was isolated from each for a total of 72 DNA isolations per strain tested. A frozen archive sample of each clone selected for DNA isolation will also be maintained for potential future analysis.

*Burkholderia mallei* and *B. pseudomallei* were alternately cultured in tryptic soy broth, followed by culturing on selective PC agar plates for a total of seven (7) passages. Bacterial DNA was isolated from the eighth passage liquid culture. At each subsequent solid media passage step, the liquid culture was plated to yield single colonies. A single colony was selected to inoculate the next liquid media passage. A portion of the colony selected for passage, along with five (5) additional colonies from each of the twelve cultures, was frozen and stored as an archive for potential future analysis (Figure 3).

### 2.3. DNA Sequencing Protocol

Single ended short read DNA sequencing was performed using a Genome Analyzer IIx (GA IIx) (Illumina, San Diego, CA, USA). Library preparation was performed using a genomic DNA sample preparation Kit. DNA clusters were generated according to the manufacturer's instructions using an Illumina cluster generation kit (Multiplexing Sequencing Primers and PhiX Control Kit v2) on an Illumina cluster station. All sequencing runs were performed with the GA IIx using the Illumina TruSeq SBS kit v5. Fluorescent images were analyzed with the Illumina CASAVA 1.8 software to obtain FASTQ-formatted sequence data of the short reads. For further details of the experimental methods, see Eppinger et al. [45].

### 2.4. Identification and Characterization of Major and Minor Components

We evaluated algorithms to process millions of short sequence reads from complex community metagenomic data. Orthogonal read alignment tools were used for comparison, GNUMap/SOAP (pipeline A), and pipeline B.

### 2.5. Selection of Assembly Software

SOAP implements the algorithm (Burrows-Wheeler transform), whereas GNUmap uses a probabilistic Needleman-Wunsch algorithm, which takes advantage of Illumina probability files to improve the mapping accuracy for lower quality reads and increase the amount of usable data produced in a given experiment. SNP calling was done under a Bayesian Inference framework as implemented in SOAPsnp by comparing an assembled genome against the reference genome used. The output files of the pipeline are a nucleotide fasta file per chromosome, a GenBank annotated file per chromosome, and a SNP file with information on all chromosomes. Performed alignment and variant analysis (SNPs and other mutations). Identified SNPs and compared relatedness of clones by building phylogenetic trees using trusted SNP variations. Trusted SNPs must have posterior probabilities >50% (Table 1).

### 2.6. SNP Accuracy and Depth of Coverage

We compared the depth of coverage in terms of the number of reads covering a particular SNP and compared that depth of coverage to the resulting posterior probability (PP) of the SNP read. The resulting analysis shows that there is a general trend of increasing PP with increasing depth.

### 2.7. Phylogenetic and Diversity Estimates

**Figure 4:**
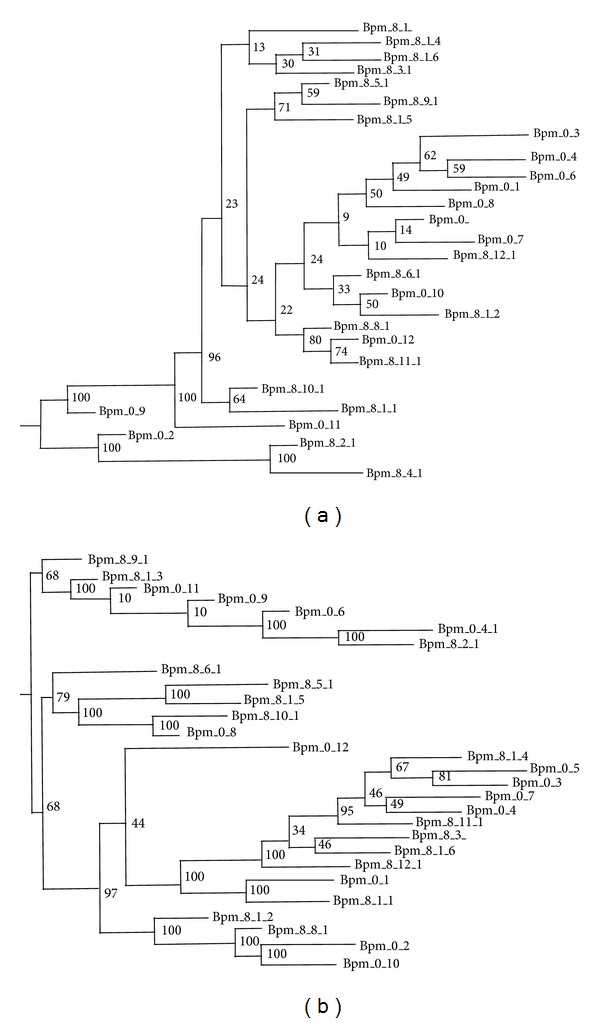
Five clones of the same lineage after passage 8 were sequenced and compared for SNPs. Posterior probabilities were calculated by the program SOAPsnp. This includes SNPs detected against the progenitor culture that was sequenced right after the first passage. (a) Illustrates chromosome 1. (b) Illustrates chromosome 2.

Phylogenetic relationships were estimated via both Maximum Likelihood (RAxML) and a network approach (SplitsTree) to estimate relationships among strains. Distance measurements are based on SNPs with posterior probabilities <95% for chromosome one and chromosome two (Figure 4).

### 2.8. Phylogenetics Is Assessed by Measurements of SNPs through Lineages

SOAPsnp takes into account base calling quality scores, as well as the number and percentage of reads receiving a particular base call at that position when calculating posterior probability scores. Theoretically, the lowest posterior probability score possible for the called base is 25% for equal probability among all bases. Posterior probabilities below 50% for an individual clone's SNP should not be trusted, unless supported by presence of the same base SNP in another clone of the same lineage.

The percent consensus column indicates consensus between accepted base calls among the different clones at that potential SNP position. The average posterior probability column indicates the average probability among clones with the particular called SNP. The Maximum Probability is an upper bound of probability that a particular SNP occurs in at least one of the five clones. It is calculated by

(1) Max⁡⁡P=1−(1−PP1)×(1−PP2)×⋯(1−PPn)×100%,

where PP_1_ is the first posterior probability for a called SNP, PP_2_ is the second posterior probability, and *n* is the number of clones possessing the called SNP. (1 − PP_*n*_) is the probability of a condition not being met for data set “*n*,” thus if there is an 80% chance of meeting an AND/OR condition in data set 1, the remaining 20% chance is multiplied by the chance of not meeting a condition in data set 2, multiplied by the percent chance of not meeting the condition in the next set and so on.

We set a SNP trusting threshold at 95% probability. If the probability values are ≥80% but <95, then it is considered probable that the SNP is present and not used in constructing phylogenies for attributing relatedness among samples or diversity.

*Pipeline B.* We trained this pipeline on *in-silico* data to validate algorithm performance on being able to distinguish bacteria down to strain level and to differentiate between highly related genomes. The process was performed in blinded fashion and genome identification was based on probabilistic values for matching criteria. Probability of obtaining matches above threshold of established criteria needs to be calculated for each matched position. The level of confidence is based on variations from reference genome sequences. Data sets possessing similar sequence variations are clustered together by phylogenetic analysis.

Pipeline B is an integrated set of public domain tools and a custom SNP calling method modified from the BFAST algorithm to find a candidate alignment position for each read. The mapping depends on a set of index masks to determine which locations in a read require matching as part of the scoring process. The aligned reads are converted to the public domain SAM format, sorted according to position along the reference and then submitted for mPileUP analysis via the Samtools software suite [11].

## 3. Results and Discussion

**Figure 5:**
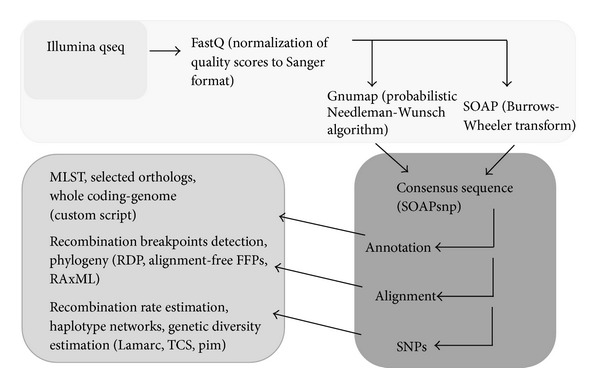
*Data Analysis Pipeline*. SOAPsnp was used to find SNPs in the data. The criteria for SNP validation in SOAPsnp is rather low. Variant validation is highly critical in metagenomic samples, more so than with homogeneous samples. False variants created by sequencer error can quickly change the results of forensics analysis in metagenomic samples where lower coverage depth and partial base consensus conditions are expected, whereas base consensus can be demanded in homogeneous sample data sets.

Sequence reads were trimmed and filtered by a custom script. Orthogonal read alignment tools were used for comparison, GNUMap/SOAP (A) and pipeline B.  Figure 5 displays our genomic data analysis pipeline A. GNUMap is better at detecting reads that differ from the reference sequence. SOAP is much faster at aligning raw reads than GNUMap, but GNUMap attains a higher percentage of aligned reads than SOAP. The differences in performance can partially be explained as speed versus accuracy tradeoffs between heuristic and probabilistic algorithms. Most other approaches such as search engines like BLAST rely on heuristic analysis to find approximate answers to questions. Heuristic approaches, however, are limited because they only identify simple alignments or lack of alignments in data, and do not guarantee that all possible matches are detected. Furthermore, since we observe a continuum of variants, the concept of characterization of samples based solely by SNP calls becomes somewhat perilous from a forensic point of view. The population structure within a single colony may arise from multiple factors, such as growth time and conditions. While we are not aware of specific quantitative analyses of how total culture time impacts population diversity, it is conceivable that the number of SNPs called based on the fraction exceeding 0.5 in frequency could indeed depend on such factors. This makes standardization of protocols a high priority for comparative analysis requiring growth in culture. It would be most advantageous to avoid any culturing step when there is sufficient available sample for direct analysis by NGS, but this, of course, cannot be guaranteed in practice.

In Table 2, we summarize specific Pipeline B alignment results for *Burkholderia pseudomallei* and *Burkholderia mallei* and *Bacillus globigii* for comparison to our nonalignment screening analysis results. As discussed above, two *Burkholderia* species had statistically high *Z* values outside the expected range for an exact match. These nonalignment results can be explained by data in the third column in the table, which shows the total fraction of nonreference base calls for reads successfully aligned to the reference genome. For *Bacillus globigii*, the baseline value of 0.018 reflects the contributions of the base calling and indel error rate of sequencing process, as well as population structure within the *Bacillus globigii* sample. For the *Burkholderia*, the nonmatching fraction is ~30% higher than *Bacillus globigii*, and this additional biological “noise” is reflected in an even higher *Z* value. While clearly our hypothesis test is sensitive to this population structure, it does tell us something useful about the sample and again highlights the importance for standardization of growth conditions, and potentially multiple calibrants to represent the population evolution tendencies of difference classes of organisms, as well as to capture potential sequence-dependent (i.e., high GC content) error processes. We note that the higher population diversity in the *Burkholderia* is also reflected in the number of SNPs called by our Pipeline B analysis.

The *Z* values are probability scores, calculated *P*(detect) for observed mean signature coverage from sequence reads. *Z* value (statistic) are derived by subtracting actual measured *P*(detect) from the interpolated value, representing this difference in units of standard deviations. *Z* values < ~2.0 given probability values <~0.01. To illustrate distribution of *Z* values obtained for unfiltered reads at mean signature coverage up to 150x, we plot values measured using within run calibration and external calibration.

The value represents the expected value from true match to a reference. The measured value (the hypothesis test) is the measurement on actual run data. To achieve accurate genome identification from populations of reads against reference database genomes using the calibration, we compute mean signature coverage for the reads against the reference, then interpolate from the calibration to estimate the predicted *P*(detect) for an observed mean signature coverage for a true match.

One final and interesting aspect of the alignment/mapping pipeline analysis is seen in the fourth and fifth columns of Table 2. While the *Burkholderia* had greater genetic diversity than *Bacillus globigii*, *B. mallei* samples had lower fractions of successfully mapped reads, and a high fraction ~3% of unmapped based positions (gaps) in the reference genome. This can be explained by the fact that *B. mallei* genomes are unusually rich in identical insertion sequences which are mobile within the genome and promote genomic rearrangements. A deeper analysis of the gaps in the reference in our case shows they exist due to algorithmic choices in use of the BFAST tool for aligning reads that have a large number of candidate locations (assign to all locations versus assigning them at random) which results in underrepresented regions [11]. This represents another algorithmic caveat when dealing with genomes with high degrees of repetition.

When trying to detect SNPs that are useful for distinguishing related strains, it is valuable to compare a broad sampling of sequence information from subspecies being studied. Herein, we sequenced twenty-eight samples from the passage experiments. Tables 3 and 4 illustrate sequence variation detected when clones from the final passage where compared to their lineage progenitors. These clones represent the diversity of the genetic populations in the original source vial. Several clones from each of the lineages were also sequenced and compared to themselves and across the bulk population. For example, samples 8_1_2 and 8_1_3 have identical genotypes for chromosome 1 as the reference (as expected), but there are also clones that have variation from the reference. This indicates the direction of diversity that specific lineages are moving in. Some SNPs are conserved across the population while others are unique. Characterization of populations is essential for attribution.

We compared sequence information for samples across all twelve lineages that were derived from a common ancestor. For example, comparison of 8_10_1 to its progenitor shows that the genotypes are the same, as is the case for sample 8_12_1. Other samples have variants that are not in their progenitor, illustrating the complexity of sequence comparison of populations from a single source. We reduced these complexities to be able to derive a process to compare samples and their populations. The comparison is two-fold: (1) comprehensive analysis across the entire genome, and (2) population-wide comparison of clones to characterize the diversity of each sample. It is on account of sample's diversity that making matches of samples based on population characterization improves accuracy of attribution.

## 4. Conclusion

Leveraging whole genome sequencing analysis strategies, entire genetic content of samples (populations) can be detected and identified as major and minor components. We have demonstrated effective analysis of lineage populations. In addition, we mapped the direction of mutation and compared taxonomic relationships and assembled genomes even when there were minor differences between genomes from the same source (see Figure 5). Using biothreat agents, *Burkholderia pseudomallei* and *Burkholderia mallei*, we cultured isolates, following mutations during passage, sequenced members of each population, built their individual genomes, and phylogenetically compared their relationships. These populations were measured and their diversity mapped with passage, which in turn enables traceability and attribution to a single source (Figure 3). We identified variation of these sample populations, indicating the unique mutations that are the DNA fingerprints of the sample profile and determined the entire population structure in the sample. We made comparisons against the genomes and direct comparison of sequence content between samples.

The diversity between passaged samples tracks with the lineages and in some cases there is overlap in genetic distance between members of the populations in different lineages, as expected (Figure 4). Overall, the pattern of diversity is spreading out from each lineage as a sphere of diversity. Hence, for forensics, the passaged material's population is dependent on the source's original content and the sequenced material is attributed back to the source. While individual members of the population are diverging and more distant phylogenetically, they are part of the sample's profile and match back to the source. Comprehensive analysis across the populations points the results (passaged samples as evidence) of each sample (even though sequence reveals that each is not 100% identical match to other members of the population) back to the single source.

We applied the *Z*-value hypothesis testing approach to “detect” the specific threat agents we sequenced in multiple runs using calibration and quality control measures. In each case, a reference sequence with the same strain name as the sequenced organism was available in the Genbank database: *Burkholderia mallei* strain China 7 (ATCC 23744) and *Burkholderia pseudomallei* strain 668. The sequences were from a single colony, which corresponds to a single constituent within the parent population (source). Generally, during growth, this bacterium will accrue new random (and fitness-selected) mutations and rearrangements that result in consensus and population differences in the sequence content with respect to the reference genome.

## 5. Mapping Reads

Table 2 illustrates alignment-based mapping statistics for threat agents and our calibrant for comparison to nonalignment *Z* values. The higher *Z* values for *Burkholderia* are reflective of the higher fraction of non-reference-matching base calls in these samples, and is indicative of greater population diversity compared to calibration. *Burkholderia* had greater SNP calls compared to *Y. pestis*. The larger unmapped base counts along the reference genomes for *Y. pestis* and *B. mallei* are due to insertion elements that are highly mobile within these genomes and promote rearrangements. In addition to sample diversity, there are sequencing errors and artifacts to account for. Unlike other groups that ignore sequencing artifacts, we account for system errors to assign candidate locations to reads when there is a high multiplicity of candidate alignments.

Pipeline B enabled appropriate relationship evaluation of the populations because these samples all contain a continuum of variants. In such circumstances, SNP calling is not terribly meaningful way to approach the problem, and methods like SOAP have inherent algorithm bias that reduces their use for relationship analyses, because SNPs which occur close to other SNPs produce low confidence calls. Our goal was to evaluate the continuum of variation (major SNPs, minor SNPs, and background noise).

There are several advantages of this approach. The calibrant tools triage the sequence data, like a funnel, to improve the processing of accurate confident data instead of using computing time on background that masks interpretation of the populations. The system error generated that noise, but it is dependent on starting sequence input; hence, variable from sample to sample, this means that millions of sequence reads from Illumina data cannot be compared from sample to sample without the calibrant.

It is clear that direct population-sequencing characterizes entire genomes as biomarkers for sample profile comparisons. However, it is important to understand the mechanisms behind probabilistic matching approaches. Herein, for example, in this case, the SNP calls, for pipeline A, are above the confidence criteria, but not called because the high number of SNPs in a small window of sequence. Hence, pipeline B provides accurate sequence information for tracking populations and matching samples to source.

If more conservative calls are made, then some SNP calls by only one algorithm could be assigned a lower confidence score. It is not so much a question as to which algorithm is more accurate as a question of the level of confidence for the specified conditions. Users need to understand the criteria they are satisfied with.

Pipeline B processed reads to establish the edge of the “background” noise, projecting a threshold to set a false call rate against this background of 0.1/genome analyzed, and then called SNPs above that threshold. Thus, for comparison purposes, how each user will choose to treat the population will determine whether the analyses will coincide. Analysis of the population contained in the sample is the key to forensic attribution. Comprehensive analysis of the genetic constituents establishes the sample profile. Profiles can be compared to determine the relatedness of the populations in the samples, not just one or two selected targets. Comprehensive analysis also strengthens the probability of the match and increases the confidence to include and exclude samples as matches.

Overall, direct sequence analysis of populations, as a biomarker, reveals the variation of genomes. Our experimental design increased homogeneity produced by single cell bottlenecks allowed a controlled analysis of the data. Many derivatives of experimental design could be extended from our work to further characterize the extent of genome variation within populations and strategies to employ to use sequencing information across entire genomes as biomarkers. With the increasing availability of complete genome sequence data from multiple microbial pathogens, comparative genomics has recently emerged as a powerful tool to understand the basic molecular properties of pathogens. Hence, population-sequencing can be used as a direct method to identify disease agents and genomes, rendering the entire genetic content, variation and diversity as the biomarker(s) for detection. Our study confirms utility of alignment-free and alignment-based approaches in defining the presence and significance of genomic variation in populations of *B. pseudomallei* and *B. mallei*. Further studies are required to determine the full extent of variability and their relationship to biological fitness in the environment and to disease pathogenesis in the human host.

Finally, sequencing analysis is highly sensitive in tracing genomic differences among isolates. Direct use of sequencing for characterization of populations of genomes present in samples provides insights into the number and types of microorganisms present. While biomarkers targeted on detection of a disease agent at the genus or species levels may indicate a threat is present, comprehensive analysis of variants, near-neighbors, and the community structure will improve the accuracy of disease detection and identification of the causal agent adding important information about disease context and community. Using DNA elements across the entire genome represents a better biomarker than PCR probes, which target limited information. Population-sequencing identifies the disease agent, provides insights on variation, and captures information about more than just a single target, indicating multiple viral and bacterial pathogens and commensals contained in samples.

## Figures and Tables

**Table 1:** Shows two genome positions analyzed by pipeline A (GNUPMap/Soap) as examples of the pipeline processing. The SNP file data analysis has 15 columns. The SNP file data includes the following: (1) reference genotype; (2) consensus genotype; (3) quality score of consensus genotype; (4) best base; (5) average quality score of best base; (6) count of uniquely mapped best base; (7) count of all mapped best base; (8) second best bases; (9) average quality score of second best base; (10) count of uniquely mapped second best base; (11) count of all mapped second best base; (12) sequencing depth of the site; (13) rank sum test *P* value; (14) average copy number of nearby region; and (15) whether the site is a dbSNP (1, yes; 0, new SNP). The quality score in field 3 is the posterior probability, and the range is between 0 and 1 (The reported score is PP × 100, so the range is 0–100). The quality score in field 5 and 9 is the average quality score of the best or 2nd best base, respectively. This corresponds to the Illumina quality scores from the original QSEQ or FASTQ files, and the range is between 0 and 40.

1	2	3	4	5	6	7	8	9	10	11	12	13	14	15
A	T	36*	T	37	4	4	A	36	3	3	7	1	1	0
A	G	99	G	38	6	6	A	0	0	0	6	1	1	0

*This example SNP is called with a posterior probability of 36%. The reference genotype is A and the consensus is T. The best base identified is T with a *q*-score of 37, while the 2nd best base is the same as reference with a *q*-score of 36. The total depth at the site is seven, with four reads supporting T and three reads supporting A. This SNP should be rejected by setting an appropriate posterior probability cutoff and not used to determine diversity or phylogenetic distance relationships.

**Table 2:** Pipeline B alignment-based mapping statistics for threat agents and our Bg calibrant for comparison to nonalignment *Z* values.

Organism	*z*-score nonmapping	Nonreference base fraction (mapped reads)	Mapped read fraction	Unmapped reference bases	SNP calls
B.mallei	3.06	0.025	0.94	166,439	431
B.pseudomallei	2.87	0.027	0.98	859	365
B.globigii*	0.02	0.018	0.99	0	0

The *Z* values for *Burkholderia* are reflective of the higher fraction of non-reference-matching base calls in these samples and is indicative of greater population diversity compared to the Bg calibrant sample. The larger unmapped base counts along the reference genomes (column 5) for Bm are due to insertion elements that are highly mobile within these genomes and promote re-arrangements. BFAST default parameters for assigning candidate locations to reads when there is a high multiplicity of candidate alignments across the reference genome resulted in these gaps. Representative mapping statistics: 10,000,000 Illumina reads, 100 bp. *Calibrant.

**Table 3:** SNP calls for *Burkholderia* pseudomallei on chromosome 1. Progenitors are labeled 0_1 to 0_12. Clones from the 8th passage are labeled 8_*x*_*y* to indicate their respective lineage (*x* = 1–12) and the number of clones selected for comparison (*y* = 1–6).

Genome position	Reference	0_1	0_2	0_3	0_4	0_5	0_8	0_10	0_11	0_12	8_1_1	8_1_2	8_1_3	8_1_4	8_1_5	8_1_6	8_3_1	8_5_1	8_6_1	8_8_1	8_9_1	8_10_1	8_11_1	8_12_1
27694	C										A			A										
30272	G				A																C			
198367	C		T	T											A		T							
208896	G		C																					
208898	G		C																					
208901	G		A																					
209253	G										A					A								
238223	G										A												A	
238226	T	A									A												A	
238248	G	A																						
254143	C																							
260159	C																	G						
309954	G			C																				
387701	T		G																					
417834	C	T																						
417940	T																	A						
439654	C	A				A					G						G							
468747	G			A							C												C	
473445	T																						G	
502149	T																							
522021	C	A																						
535552	G			C																				
575316	C				A																			
575340	T		A																					
636702	T																			G				
637354	C																	A						
637366	T										C								C					
719754	G		A																					
748773	T									C														
748778	G										A													
752001	G			A																				
755855	G													C										
827221	G										A			A							A			
830204	C																							
840516	C										T													
846684	C										T			T										
856204	G										A													
856229	G										T							T						
867860	T			G																				
879922	G	A																						
908149	C										G						G							
918155	G				A																			
918163	G				A																			

**Table 4:** SNP calls for *Burkholderia* pseudomallei on chromosome 2. Progenitors are labeled 0_1 to 0_12. Clones from the 8th passage are labeled 8_*x*_*y* to indicate their respective lineage (*x* = 1–12) and the number of clones selected for comparison (*y* = 1–6).

Genome position	Reference	0_1	0_2	0_3	0_4	0_5	0_8	0_10	0_11	0_12	8_1_1	8_1_2	8_1_3	8_1_4	8_1_5	8_1_6	8_3_1	8_5_1	8_6_1	8_8_1	8_9_1	8_10_1	8_11_1	8_12_1
16302	C										A			A										
63867	T										C	C												
112549	A				G																			
112589	A				G																			
125529	T										C						C							
153871	C										A		A											
255127	C		T	T	T						T						T			T				
257976	A																							
289329	T																							
334349	G										A					A								
371920	G										A												A	
401983	C										T			T										
429628	G				A																			
438505	A										G											G		
446580	T																							
479883	T													G										
482145	G		A																					
563692	A										G			G										
568009	G										A	A												
656370	C				A																			
673008	G			T							C						C			T			T	
767675	C										T				T									
767835	T										C							C						
769495	C			A																				
770544	C	A																						
790672	C										A							A						
793808	G										A		A											
794151	G										A		A											
794210	G										A													
794352	G																						T	
794608	T																	G						
794942	G										A											A		
821535	G		A																					
859546	A										G			G										
859585	G										T			T										
